# Oxidative Stress and Male Reproductive Health—First Edition

**DOI:** 10.3390/antiox15060700

**Published:** 2026-06-01

**Authors:** Robert J. Aitken, Joel R. Drevet, Zamira Gibb, Geoffry N. De Iuliis

**Affiliations:** 1 Centre for Reproductive Science, University of Newcastle, Newcastle, NSW 2308, Australia; zamira.gibb@newcastle.edu.au (Z.G.); geoffry.deiuliis@newcastle.edu.au (G.N.D.I.); 2 Reproductive and Family Health Program, Hunter Medical Research Institute, New Lambton Heights, Newcastle, NSW 2305, Australia; 3 Institut de Génétique, Reproduction et Développement (iGReD), Faculté de Médecine, Université Clermont Auvergne, CRBC, 28 Place Henri Dunant, 63001 Clermont-Ferrand, France; joel.drevet@uca.fr; 4 Évaluation de la Semence Mâle (EVALSEM), Clermont Auvergne Innovation, Faculté de Médecine, Université Clermont Auvergne, CRBC, 28 Place Henri Dunant, 63001 Clermont-Ferrand, France

Over the past half-century, the world has witnessed a dramatic decline in human fertility, which began in the 1960s and has continued unabated ever since [1]. The etiology of this global decrease in fertility rates is complex and involves an intricate mixture of cultural, socioeconomic, biological, clinical, and environmental factors affecting both male and female reproduction [2]. While emphasis is traditionally placed on the dwindling desire of young couples to have children, more worrying is emerging evidence suggesting that our species is experiencing a decline in fecundity, i.e., our fundamental capacity to reproduce—even if the motivation to have a family is present [3].

Among the potential reasons for this fall in fecundity are clear indicators of rising male infertility. Across the world, average sperm counts are in decline, as are other attributes of semen quality, including progressive motility and sperm morphology [4,5,6,7,8,9,10,11,12]. Adding fuel to the fire of a male fertility crisis is the incidence of testicular cancer, which has risen to become one of the commonest cancers in young men, particularly in Europe [13]. Although the quality and consistency of data on diminished fecundity might be far from perfect, the general consensus is that the global prevalence of male infertility is increasing and that this trend is particularly evident in the world’s most socioeconomically advanced nations [14]. This change in reproductive competence is being driven by a range of factors associated with modern society, including advanced paternal age at conception [15], lifestyle factors such as smoking, diet and obesity [16,17,18], environmental toxicants including microplastics [19], heavy metals [20], endocrine-disrupting chemicals (for example, bisphenol A [21], phthalate esters [22], poly- and perfluoroalkyl substances [23]), pharmaceutical contaminants such as parabens [24,25], and aspects of climate change, particularly heat [26]. Although the array of factors apparently affecting male fertility may seem bewilderingly large, the one thing that they all share is the capacity to induce a state of oxidative stress.

The notion that oxidative stress might be associated with male infertility has a long history stretching back to the pioneering studies of John McLeod [27], who, as early as 1943, demonstrated the positive impact of catalase on human sperm motility, thereby implicating the metabolic generation of hydrogen peroxide in the senescence of these cells in vitro. The harmful effects of oxidative stress, and specifically lipid peroxidation, on human sperm function were subsequently highlighted by Thaddeus Mann and Roy Jones at the University of Cambridge in the late 1970s [28]. By 1987, the Alvarez and Aitken groups had independently established a clear link between defective sperm function and the excessive generation of reactive oxygen species (ROS) [29,30].

The fundamental importance of such stress in the etiology of defective sperm generation and function is now a well-accepted paradigm [31]. During the early stages of spermatogenesis, the structural and functional integrity of the male germline is maintained more by the deletion of defective cells than by the instigation of error-prone DNA repair pathways. So efficient is this process that 80% of all male germ cells are thought to meet an apoptotic/ferroptotic fate, during which mitochondrial ROS generation and lipid peroxidation play a key role in the orchestration of cell death [32,33,34]. If the germ cells survive this meiotic checkpoint and differentiate into haploid spermatozoa, their capacities for repair or apoptosis are greatly curtailed. The vulnerability of these cells is compounded by the limited volume and distribution of cytoplasm in which to house enzymes involved in cellular defense and repair. In addition, these cells contain abundant targets for free radical attack, including unsaturated fatty acids, nucleic acids (DNA and RNA), thiol-rich proteins, and sialylated glycoproteins. As a result, spermatozoa are very prone to accumulating the products of oxidative damage, including lipid hydroperoxides [35], aldehyde-adducted proteins [36], advanced glycation end products [37,38], and oxidative DNA damage in the form of 8-hydroxy-2′-deoxyguanosine (8OHdG) [39].

Given this inherent vulnerability, it is perhaps surprising that ROS play such an important physiological role in sperm biology. Thus, the oxidative polymerization of glutathione peroxidase molecules in the mitochondrial sheath, as well as the crosslinking of protamines in sperm chromatin, stabilizes and protects these structures as spermatozoa differentiate and mature within the male reproductive tract [40]. Furthermore, following ejaculation, ROS are involved in both sperm capacitation and fertilization through the abilities of these metabolites to drive cAMP generation, silence protein phosphatase activity, activate proteolytic enzymes, and influence the redox status of transition metals at the active sites of many enzymes [41]. These cells therefore live their lives on a knife-edge. A low level of sustained ROS generation is required to not only fashion the physical architecture of these cells as they differentiate in the male tract but also support their subsequent activation during ascent to the site of fertilization in the female tract. Throughout their lifespan, spermatozoa maintain a state of redox balance by virtue of intracellular defensive enzymes, particularly peroxiredoxin-6 (PRDX6) [2], as well as a collection of powerful antioxidants that are present in the extracellular space. Thus, seminiferous tubule fluid, epididymal plasma, seminal plasma, and uterine fluids are all well-endowed with antioxidant enzymes and free radical scavengers [42]. However, if the exposure to/generation of ROS exceeds the defensive capacity of this localized pool of antioxidants, a state of oxidative stress is induced. The latter compromises the fertilizing capacity of the spermatozoa and, if conception should occur, the health and wellbeing of any offspring they generate. Importantly, free radical attacks on the male genome are not random but affect some chromosomes more than others. In the mouse, chromosome 19 is most adversely affected, whereas in human spermatozoa, it is chromosome 15 [43,44]. This is clinically significant because genetic and epigenetic changes in this particular chromosome are associated with a range of neuropsychiatric conditions that are strongly correlated with the age of the father at the moment of conception (e.g., autism, spontaneous schizophrenia, bipolar disease, and Marfan syndrome) [45].

Awareness of the importance of oxidative stress as a major contributor to male infertility has grown dramatically over the last 25 years, such that there are now thousands of papers published on this topic. Because the field is advancing so rapidly, it is critical that we take stock of recent developments that not only further our understanding of the underlying pathophysiology but also highlight the opportunities such knowledge presents for improved methods of diagnosis and treatment.

Here, we have brought together a collection of papers that capture recent advances in our understanding of the role of oxidative stress in defining male reproductive function. A majority of the papers focus on fertility and have a clinical orientation. However, one article on equine reproduction has also been included to emphasize that such stress is a key contributor to male infertility across all mammalian species. In addition, this Special Issue also features a paper on priapism to underscore the importance of redox chemistry in other male reproductive pathologies, not just infertility. Altogether, this collection of articles features five extensive reviews that provide a clear overview of where this field is currently positioned, followed by five original articles describing recent insights into the redox regulation of male reproductive function.

The collection is introduced by an insightful overview of the mechanisms by which oxidative stress impacts male reproductive health, with a particular focus on the role of antioxidants in treating this condition (Contribution 1). There is a clear rationale for the use of antioxidants to treat male infertility; however, clinical trials designed to explore the therapeutic potential of such treatments have largely yielded disappointing results [46]. There are many reasons for this. First and foremost, the authors point out that we cannot initiate clinical trials until effective methods have been developed for diagnosing this condition in a clinical setting. The indiscriminate use of antioxidants to treat males who may not be suffering from oxidative stress risks inducing a state of reductive stress [47]. As a consequence of poor patient selection, any therapeutic benefit potentially derived from antioxidant supplementation will just be lost in the noise. At present, we have no solution to this problem: chemiluminescent measurements cannot be calibrated and are heavily affected by leukocyte contamination [48], the MiOXSYS system does not detect ROS generation by spermatozoa and has been clinically criticized [49], and, while flow cytometry methods are effective, the infrastructure required to make such measurements are beyond the reach of most clinical environments [50]. In addition, our understanding of which antioxidants to use to treat specific causes of oxidative stress, in terms of bioavailability, mechanism of action, duration of treatment, dose, formulation, and safety, is still rudimentary. The authors suggest a potential combination of antioxidants, based on an extensive review of the available evidence. However, they also stress that we are still a long way from realizing the evident potential in this field. Unfortunately, andrologists possess neither the diagnostic tools nor the biochemical/pharmacological knowledge to generate robust guidelines on how antioxidants might be used to address male infertility precipitated by oxidative stress. We can see the promised land, but it remains frustratingly distant.

A novel approach to finding diagnostic markers for oxidative stress in the male germline would be to study redox-mediated changes in the protein complement of these cells. The well-recognized vulnerability of spermatozoa to electrophilic attack demonstrates that the redox status of protein thiols is of central importance to the functional biology of these cells. While most considerations of this topic simply focus on thiol oxidation, there are many other forms of potential modification (S-nitrosylation, sulfhydration, glutathionylation, CoAlation, and carbonylation) that are potentially just as important, as highlighted in Contribution 2. This insightful review emphasizes the reversibility of many of these modifications and their potential physiological role in protecting sulfhydryl groups, so that ‘thiol switching’ can be deployed as a regulatory mechanism. The importance of such switching strategies explains why the irreversible, covalent modification of protein thiols by electrophilic aldehydes (such as 4-hydroxynonenal), generated as a by-product of lipid peroxidation, has such a devastating impact on sperm biology. Thiol switching is particularly important for controlling protein phosphatase activity and, thus, the phosphorylation-dependent signal transduction cascades that regulate key biological events such as capacitation and acrosomal exocytosis. The protection of protein thiols from premature or irreversible oxidation involves the scavenging activity of enzymes, notably PRDX6, and the formation of reversible thiol complexes, notably via S-nitrosylation, sulfhydration, and CoAlation. The latter has emerged as a particularly important regulator of sperm capacitation. In non-capacitated spermatozoa, CoAlation levels are high, and thiol availability is suppressed. However, the induction of capacitation is associated with a reduction in protein CoAlation, rendering the uncovered thiols susceptible to oxidation and enabling the activation of molecular switches controlling protein phosphatase activity. Such deep insights into the redox life of spermatozoa open up new opportunities for the development of biomarkers for redox imbalance in spermatozoa and suggest precision therapeutic strategies, tailored to whichever pathways are disrupted.

The loss of sperm function following cryopreservation is an excellent example of a clinically important process that is vulnerable to oxidative stress (Contribution 3). Current methodologies are associated with impaired sperm motility and a significant increase in the levels of DNA damage sustained by these cells [51]. The realization that ROS are of central importance in the etiology of cryoinjury has triggered a plethora of studies into the use of antioxidants to improve semen quality following cryopreservation [52]. This is a greater challenge than one might imagine. While oxidative stress is a major factor in the induction of DNA damage during cryopreservation, the freezing process also exposes the spermatozoa to damage due to ice crystal formation as well as osmotic and thermal shock. Furthermore, finding antioxidants that will curtail oxidative damage while allowing the redox regulation of sperm physiology to proceed unhindered is a difficult task. Nevertheless, the more we understand about the sources of oxidative stress and the intracellular targets for ROS attack, the better able we shall be to design media and protocols that optimize sperm functionality following cryopreservation.

While attention is traditionally focused on oxidative stress in spermatozoa, this Special Issue also contains a unique transcriptomic analysis of human spermatogonial stem cells (SSCs), which are no less dependent on their redox status [53,54] (Contribution 4). This bioinformatic tour de force highlights the fundamental importance of antioxidant gene expression during spermatogenesis, from the earliest commitment of SSCs to the spermatogenic pathway to the modeling of gametes during spermiogenesis. However, the expression of oxidative stress-related genes during germ cell development is not constant; it is dynamically modulated, with higher levels of activity during post-meiotic germ cell development, when cytoplasmic space is becoming restricted, and the germline’s intrinsic repair machinery is closing down.

In another technical tour de force, double-knockout (DKO) mice, lacking glutathione peroxidases 4 and 5 (*snGpx4^−/−^*; *Gpx5^−/−^*), were used to create a genetically engineered model of oxidative stress in the male germline. The spermatozoa of such animals were shown to possess significantly elevated levels of 5-hydroxymethylcytosine (5hmC) in concert with high levels of 8OHdG, confirming the profound impact of oxidative stress on the epigenetic landscape in this DKO model (Contribution 5). Antioxidant therapy was successful in reducing the elevated expression of 5hmC but curiously increased this biomarker in wild-type control mice. When the analysis was focused on CpG islands using the RREM-seq technique, oxidative stress was shown to be associated with a decrease in methylated DNA, while hydroxymethylation was increased compared with wild-type spermatozoa. A possible explanation for this situation is that under conditions of oxidative stress, the presence of 8OHdG adducts within CpG islands impedes methylation as a result of steric hindrance and allosteric inhibition of DNA methyltransferase activity. On the other hand, for CpG cytosines that are already methylated, the presence of 8OHdG recruits OGG1 (8-oxoguanine DNA glycosylase), which initiates the base excision repair (BER) pathway to excise the damaged base. This BER machinery creates transient single-strand breaks and recruits chromatin-remodeling factors to the CpG island. This repair environment facilitates the recruitment or activation of TET (Ten-Eleven Translocation) enzymes that actively oxidize adjacent 5-methylcytosine (5mC) into 5-hydroxymethylcytosine (5hmC), leading to enhanced hydroxymethylation [55,56,57]. This study highlights the very dynamic nature of epigenetic modifications to the male genome and its vulnerability to cellular redox status. Given the undeniable importance of sperm epigenetics in programming embryonic development, antioxidants should clearly be used with care in the treatment of male infertility.

In this context, it is particularly important that antioxidants are administered only to males experiencing infertility associated with oxidative stress. Moreover, even when oxidative stress is identified, we should be careful to tailor the dose of antioxidant administered to the level of oxidative stress observed. In addition, we should aim to discontinue antioxidant treatment once redox balance has been restored, thereby avoiding the risk of over-supplementation and the development of reductive stress. In order to facilitate this strategy, the field needs a simple, point-of-care method for detecting oxidative stress in semen. In Contribution 6, a methodology is described that lays the groundwork for just such a point-of-care assay that correlates well with semen quality [58].

With the help of such diagnostic tools, we should be in a position to explore the multifarious causes of oxidative stress in the male germline. In this vein, a key contributor that is particularly important in socioeconomically advanced countries is obesity [59,60]. The precise basis of the relationship between obesity and oxidative damage in spermatozoa has, however, remained a mystery. In Contribution 7, a possible mechanistic link is proposed in the form of deoxycholic acid (DCA), a metabolic by-product of intestinal bacteria linked to diets rich in saturated fats. In this study, direct exposure of human spermatozoa to DCA was found to stimulate mitochondrial ROS generation, enhance lipid peroxidation, and induce oxidative DNA damage. These redox changes were found to suppress the high levels of capacitation, hyperactivation, and acrosomal exocytosis observed following exposure to fetal cord serum ultrafiltrate, without significantly impacting either total or progressive motility. These results indicate that sperm function can be oxidatively impaired, even when sperm motility is normal [61]. Therefore, using sperm motility as an isolated selection criterion for oxidative stress may be fundamentally flawed.

Outside of dietary factors, there are many other external contributors to oxidative stress in the male germline, including smoking, exposure to environmental pollutants, heat, radiofrequency electromagnetic radiation, drug and alcohol abuse, strenuous exercise, and psychological stress [62]. In addition, this Special Issue highlights the importance of another factor—night shift work (Contribution 8). The latter was found to be an independent predictor of oxidative stress associated with disruptions of semen quality, particularly progressive motility, increased sperm DNA damage, and subfertility [63,64]. Importantly, antioxidant therapy was shown to significantly improve semen quality in workers suffering from the disruptions precipitated by shift work. These results open the door to gold-standard, prospective, randomized, blinded control studies to establish the value of antioxidant therapy in this group of workers.

The impact of oxidative stress on male reproductive function is not confined to the germline. Leydig cells, for example, are vulnerable to free radical attack and are implicated in the age-related decline in circulating testosterone levels [65]. In the penultimate article in this series (Contribution 9), the authors consider the role of oxidative stress in priapism—a painful penile disorder that is particularly prevalent in cases of sickle cell anemia. This condition features a complex etiology, a central element of which is the ability of superoxide anion (O_2_^−●^) to scavenge nitric oxide (NO) and disrupt the orderly regulation of penile blood flow during the erectile response. Given the importance of oxidative stress in the development of priapism, it is not surprising that antioxidants such as resveratrol have been found to possess some therapeutic value [66]. Moreover, the authors propose the idea of combining an antioxidant such as resveratrol with an NO donor to optimize blood flow while inhibiting oxidative stress [67]. The use of such novel antioxidants in combination with established sickle cell therapies holds considerable promise for the integrated clinical management of these comorbidities in the future.

Finally, the impact of oxidative stress on male reproductive function is also not confined to humankind. Redox imbalance is known to impact the fertilizing potential of spermatozoa in a diverse range of species, from flies [68] to thoroughbred horses [69]. In the latter, the highly progressive sperm motility characteristic of this species is heavily dependent on a complex array of redox reactions involving the sperm mitochondria. Inevitably, the intense metabolic demands placed upon these impressively motile cells lead to electron leakage and the generation of ROS [70]. A detailed and elegant account of sperm metabolism in this species (Contribution 10) highlights the importance of mitochondrial dysfunction in the genesis of oxidative stress. It also emphasizes the way in which the high concentration of glucose typical of commercial semen extenders can exacerbate this problem, creating cellular damage that shares many features with the detrimental impact of hyperglycemia in diabetic patients.

The scientific papers published in this Special Issue provide a unique and powerful insight into the role of oxidative stress in the control of male reproduction. ROS are involved in the physiological regulation of virtually every aspect of the reproductive process, including spermatogenesis, epididymal maturation, fertilization, testosterone production, and normal erectile function. However, these same molecules are also involved in many pathologies that impact the male reproductive system, including failed fertility, endocrine insufficiency, erectile dysfunction, priapism, genital tract infection, and reproductive malignancies. Male reproduction is therefore a typical example of a physiological system that is subject to the Janus face of ROS—biologically essential when generated in small amounts but biologically devastating when production overwhelms the local provision of antioxidant defenses. Continued research into the duality of redox regulation will help us manage the impact of oxidative stress on male reproduction so that we can mitigate its effects rather than fall victim to its potent pathological consequences.

## Data Availability

No new data were created or analyzed in this study. Data sharing is not applicable to this article.

## Abbreviations

The following abbreviations are used in this manuscript:

8OHdG	8-hydroxy-2′-deoxyguanosine
ROS	Reactive oxygen species
DKO	Double knockout
CpG	Cytosine-phosphate-Guanine
BER	Base excision repair
TET	Ten-eleven translocation
OGG	8-oxoguanine DNA glycosylase
DCA	Deoxycholic acid
NO	Nitric oxide
O_2_^−●^	Superoxide anion
